# Advanced pancreatic cancer with long-term recurrence-free survival after radical pancreatic resection and subsequent resection of lung metastases twice: A case report

**DOI:** 10.1016/j.ijscr.2023.108724

**Published:** 2023-08-30

**Authors:** Kenjiro Hirai, Jun Takeshima, Jun Ichikawa, Asami Okabe, Hidenori Ohe, Akira Mitsuyoshi

**Affiliations:** a Department of Surgery, Japanese Red Cross Otsu Hospital, 1-1-35 Nagara, Otsu City, Siga 520-8511, Japan; b Department of Surgery, Otsu City Hospital, 2-9-9 Motomiya, Otsu city, Shiga 520-0804, Japan

**Keywords:** Isolated lung metastasis of pancreatic cancer, Lung resection, Long-term survival

## Abstract

**Introduction and importance:**

Reports on lung resection for recurrence with lung metastases after the surgical treatment of pancreatic cancer have been sporadic, and limited information is currently available on the long-term postoperative course. Furthermore, the significance of the surgical resection of recurrent/metastatic lesions after the resection of pancreatic cancer has not been sufficiently established. We herein present a long-term recurrence-free survivor after perioperative chemotherapy and pancreatic resection for primary pancreatic body cancer who underwent resection for isolated lung metastases twice.

**Case presentation:**

A 66-year-old woman with locally advanced pancreatic cancer accompanied by invasion of the splenic artery underwent distal pancreatectomy with celiac axis resection following preoperative S1 + gemcitabine therapy. Recurrence with lung metastasis was detected 42 and 62 months after resection of the primary lesion, and lung resection was performed both times. As postoperative adjuvant therapies, S1 + gemcitabine therapy was performed after lung resection. The patient has survived free of recurrence for 11 years after resection of the primary lesion and 5 years and 9 months after the second lung resection.

**Clinical discussion:**

A long interval from resection of the primary lesion to the occurrence of lung metastases and the high responsiveness of the patient to chemotherapy may have contributed to her long-term survival.

**Conclusion:**

This case suggests that if lung metastasis occurring after radical resection of the primary lesion is resected without remnants, aggressive multidisciplinary treatment, including surgical resection with the appropriate selection of cases, may contribute to improvements in patient outcomes.

## Highlights

• Reports on lung resection for recurrence with lung metastases after the surgery of pancreatic cancer have been sporadic.
• We herein present a long-term recurrence-free survivor after surgery who underwent resection for lung metastases twice.
• This case suggests that aggressive multidisciplinary treatment may contribute to improvements in patient outcomes.

## 1 Introduction

Although improvements have been achieved in the surgical treatment of pancreatic cancer due to the recent development of multidisciplinary treatment, the curative resection rate remains low. Even with curative resection, postoperative metastasis/recurrence is often observed, resulting in a poor prognosis [1]. Reports of lung resection for recurrence with lung metastases after the surgical treatment of pancreatic cancer have been sporadic, limited information is currently available on the long-term postoperative course, and the significance of the surgical resection of recurrent lesions has not been sufficiently established [[1], [2], [3]]. Furthermore, lung metastasis after surgery for pancreatic cancer is often accompanied by lymphangitic carcinomatosis and multiple lung metastases, and few patients have indications for lung resection [4]. We herein describe a patient with pancreatic body cancer who was treated by pancreatectomy after preoperative chemotherapy and subsequently underwent pneumonectomy twice with postoperative adjuvant chemotherapy for isolated lung metastases. The patient has survived in a recurrence-free state for 5 years and 9 months after the second pneumonectomy. We also review the literature. This case report has been reported in line with the Surgical CAse REport (SCARE) guidelines [5].

## 2 Presentation of case

The patient was a 66-year-old asymptomatic woman with a history of laparoscopic cholecystectomy for gallstones in 2005. Her family history was unremarkable.

In January 2011, a low echoic mass measuring 40 × 20 mm was detected in the pancreatic body by ultrasound endoscopy, and the patient was referred to our department for a detailed evaluation and treatment.

**Fig. 1:**
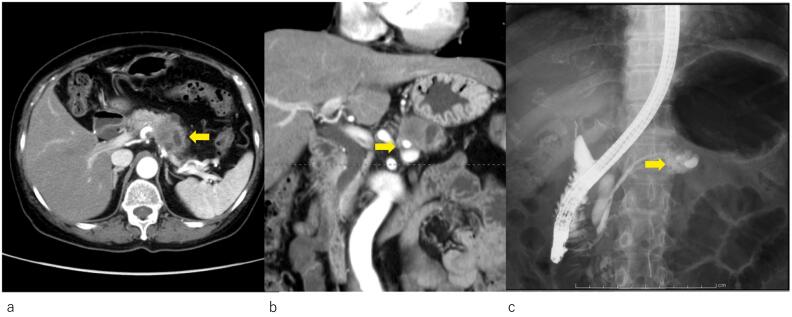
Images of the primary lesion a) Abdominal contrast-enhanced CT. A tumor shadow was noted in the distal pancreas (arrow). b) Abdominal contrast-enhanced CT. Infiltration to the splenic artery and vein was suspected (arrow). c) Endoscopic retrograde cholangiopancreatography. The main pancreatic duct was dilated to a maximum diameter of 3.2 mm, and its image terminated in the pancreatic body (arrow).

Computed tomography (CT) scans revealed a tumoral lesion in the distal pancreas, and invasion of the splenic artery and vein was suspected (Fig. 1a, b).

ERP showed dilation of the main pancreatic duct to a maximum diameter of 3.2 mm. The image of the pancreatic duct terminated in the body and was not visualized in the tail (Fig. 1c). A diagnosis of class V adenocarcinoma was made by pancreatic duct brushing and pancreatic juice aspiration cytology.

**Fig. 2:**
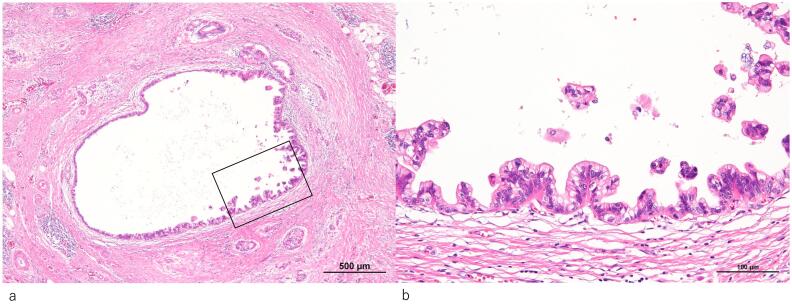
Histopathological features of resected specimens of the primary lesion (Hematoxylin-eosin stain) Non-invasive adenocarcinoma showing marked atypia was observed in the branch duct epithelium. Collagen fibers proliferated around the mass and showed marked fibrosis (a,b). (b: Enlargement of the rectangular area in a).

**Fig. 3:**
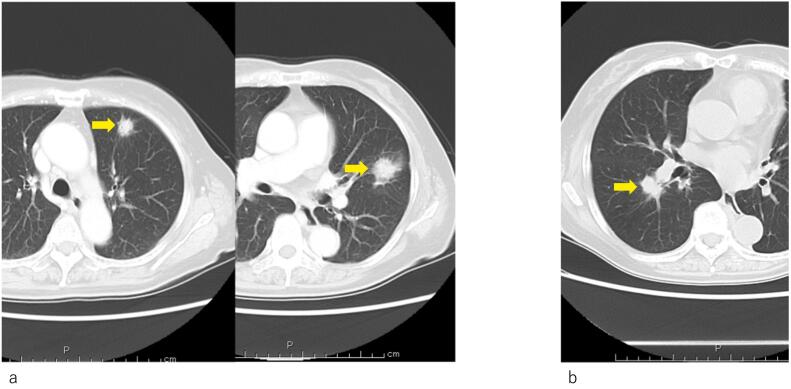
Thoracic contrast-enhanced CT images a) Forty-two months after resection of the primary lesion. Two nodular shadows were observed in the left lung (arrow). b) Sixty-two months after resection of the primary lesion. One nodular shadow was noted in the right lung (arrow).

**Fig. 4:**
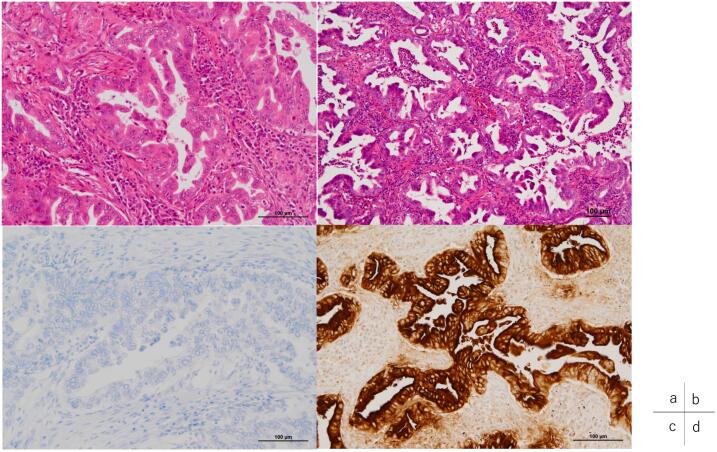
Histopathological features of resected lung specimens a) Recurrent lesion: An atypical epithelium consisting of a high columnar epithelium with enlarged nuclei proliferated, showing irregular ductal structures (Hematoxylin-eosin stain). b) *Re*-recurrent lesion: Atypical gland ducts similar to those in the primary and recurrent lesions proliferated (Hematoxylin-eosin stain). c) Recurrent lesion: Negative for thyroid transcription factor-1 staining. d) Recurrent lesion: Positive for CA19–9 staining.

Based on these findings, the lesion was diagnosed as cT3 (Asp) N0 M0 cStage IIA (UICC, 7th edition). As preoperative treatments, coil embolization of the common hepatic artery and 4 courses of S1 + gemcitabine (GS) therapy (GEM: 1300 mg day 1,8, S1: 100 mg/day, 2-weeks-on and 1-week-off) were performed, followed by distal pancreatectomy with celiac axis resection. The histopathological diagnosis was non-invasive adenocarcinoma, Pbt, 7.7 cm, TS4, ductectatic type, ypTis, pCH(−), pDU(−), pS(−), pRP(−), pPV(−), sA(−), pPL(−), sOO(−), ly0, v0, ne0, pPCM(−), pDPM(−), pN0, M0 (Fig. 2a, b). Mass shadows suggestive of metastasis were detected in the left lung (S3, S4) by CT 42 months after resection of the primary lesion (Fig. 3a); however, since it was not possible to reach a diagnosis by bronchoscopy, cytology, or biopsy, laparoscopic partial left upper lobectomy was performed. In a histopathological evaluation, the histological profile resembled that of the primary pancreatic cancer, thyroid transcription factor-1 was negative, and CA19–9 was positive; therefore, lung lesions were diagnosed as the recurrence of pancreatic cancer (Fig. 4a, c, d). Although 7 courses of GS therapy were performed after pneumonectomy, a mass was detected at S6 of the right lung by CT 62 months after resection of the primary lesion (Fig. 3b), and right lower lobectomy was performed. In a histopathological evaluation, histological features resembled those of the previous pancreatic cancer and lung metastasis; therefore, the mass was diagnosed as lung metastasis of pancreatic cancer (Fig. 4b). S1 therapy has been continued since the second pneumonectomy for 5 years and 9 months, and the patient has survived free of recurrence.

## 3 Discussion

Although improvements have been achieved in the surgical treatment of pancreatic cancer due to the recent development of multidisciplinary treatment, the curative resection rate remains low. Even with curative resection, postoperative metastasis or recurrence is often observed, resulting in a poor prognosis. The recurrence of pancreatic cancer as metastasis is regarded as a systemic disease even when the metastatic recurrent lesion is solitary or micrometastasis, and since it is generally not an indication for surgical resection, it is primarily treated by systemic chemotherapy. Median survival times (MST) after the resection of liver metastasis and the peritoneal dissemination of pancreatic cancer were previously reported to be 11.4 and 12.9 months, respectively, which are similar to those for chemotherapy for unresectable recurrent pancreatic cancer; therefore, the significance of resecting the metastases of pancreatic cancer is not considered to be sufficient [3,6].

Cases of the successful resection of recurrent lesions have been sporadically reported. According to Arnaoutakis et al., among 31 cases of recurrence with lung metastasis after the surgical treatment of pancreatic cancer, the outcomes of 9 patients who underwent pneumonectomy (MST: 18.6 months) were more favorable than those of 22 who did not (MST: 7.5 months) [7]. Thomas et al. evaluated 21 cases that underwent surgical resection for the postoperative recurrence of pancreatic cancer and showed that outcomes were more favorable for those with the resection of a single lung metastasis (MST: 92.3 months) than those with the resection of liver metastases (MST: 32.5 months). In addition, the outcome of the resection of metastatic lesions was more favorable when metastasis appeared with a disease-free interval (DFI) of 20 months or longer after the initial surgery than with a DFI of <20 months (MST: 92.3 vs. 31.3 months), and they concluded that cases of a single lung metastasis with a DFI of 20 months or longer are good indications for the resection of recurrent lesions of pancreatic cancer [8]. In addition to cases of the resection of single metastatic lesions, a previous study reported a patient who survived without recurrence for 66 months after the resection of bilateral lung metastases [9]. These findings suggest the potential of surgery as a treatment option regardless of the number or location of lung metastases if metastases are confined to the lungs and if all lesions may be completely resected.

The effectiveness of the resection of re-recurrent lung metastases has also been reported. Yasuda et al. described a case that underwent the resection of re-recurrent lung metastasis detected 5 months after pneumonectomy for the first lung metastasis following surgery for pancreatic cancer and survived for 32 months after the second pneumonectomy [4]. Since our patient has survived without recurrence for 5 years and 9 months following the resection of lung metastasis detected 20 months after the initial pneumonectomy, the re-resection of lung metastases that appear after pneumonectomy may be effective in some patients. However, the long-term follow-up and accumulation of cases are necessary to establish the indications and usefulness of the surgical treatment of lung metastases of pancreatic cancer.

According to Thomford's principles, the indications of surgery for metastatic lung tumors are as follows: (1) the primary lesions are controlled, (2) there is no extrapulmonary metastasis, (3) lung metastases are localized unilaterally, and (4) the patient tolerates surgery [10]. The case presented here fulfilled these criteria, and surgical treatment was considered to have been appropriate even if the diagnosis of metastasis of pancreatic cancer had been made preoperatively.

Distinguishing between pancreatic cancer with lung metastasis and primary lung adenocarcinoma relies on tissue similarity and immunohistochemical examinations. Concerning immunohistochemical tests, Stenhouse et al., have reported that the TTF-1 staining positivity rate was 0 % in 106 cases of metastatic lung tumors, while it was 75 % in primary adenocarcinomas, indicating the utility of TTF-1 staining in their differentiation [11]. Additionally, the CA 19–9 positivity rate in metastatic tumors has been reported as 32 % for lung cancer and 85 % for pancreatic cancer [12]. In this case, the confirmed tissue similarity with the initial pancreatic cancer surgery, coupled with negative TTF-1 staining and positive CA19–9 staining, led to the diagnosis of pancreatic cancer with lung metastasis.

While pancreatic cancer metastasizes to various organs, including the liver, lymph nodes, lung, bone, and peritoneum, it most frequently metastasizes to the liver because the pancreas is a portal-drained viscus in which cancer recurs early [13]. Many recurrent cases have accompanying distant metastatic lesions and an extremely poor prognosis. However, according to Aiura et al., autopsies of patients who survived for a long time after surgery for pancreatic cancer showed that many had lung metastases [14], and only a few rare cases developed lung metastases without liver or lymph node metastasis or peritoneal dissemination, similar to the present case. A previous study reported that lung recurrence was more frequently detected than liver recurrence or local recurrence 5 years or more after the resection of pancreatic cancer [15]. Possible routes of metastasis to the lung are the direct entry of cancer cells into the greater circulation in cases that develop retropancreatic infiltration or lymph node metastasis and the independent formation of metastatic lesions by cancer cells that have escaped the hepatic vascular bed and are captured by the pulmonary circulation in cases of isolated metastasis. Although the reason why isolated lung metastases occur later than intra-abdominal recurrence remains unclear, pancreatic cancers that innately develop isolated lung metastases appear to have milder biological malignancy and are slow growing or are highly responsive to chemotherapy [2].

In perioperative chemotherapy for pancreatic cancer, GEM and S1 are effective both pre- and post-operatively [[16], [17], [18], [19], [20]], and we also treated the present case with these key drugs alone or in combination. Regarding the treatment response, although the preoperative diagnosis was cT3, the histopathological diagnosis with resected specimens was non-invasive carcinoma. Pulmonary re-recurrence occurred after the end of GS therapy following surgery for the first pulmonary recurrence, and the patient has remained free of recurrence for 5 years and 9 months, during which S1 therapy has been continued. The high responsiveness of our patient to chemotherapy appears to have contributed to long-term survival. In addition to the present case, the effectiveness of multidisciplinary treatment for recurrence with distant metastasis after surgery for pancreatic cancer has been demonstrated. Sato et al. reported a case in which lung metastasis that developed after surgery for pancreatic cancer disappeared due to chemotherapy, and brain metastasis that subsequently occurred was treated by surgical resection and radiation therapy, after which the patient survived without recurrence for 8 months [21]. Since long-term survival has been achieved with a combination of systemic chemotherapy and the local resection of recurrent lesions, it may be reasonable to consider aggressive treatment, even for postoperative recurrence.

We encountered a patient who underwent the resection of lung metastasis twice 42 and 62 months after surgery for pancreatic cancer, received postoperative adjuvant chemotherapy, and has survived without recurrence for 5 years and 9 months after the second pneumonectomy. If lung metastasis of pancreatic cancer with a DFI of ≥20 months is resectable without residual cancer, an aggressive evaluation of indications for surgical resection and the continuation of postoperative adjuvant chemotherapy may contribute to improvements in patient outcomes. Furthermore, to avoid missing opportunities for surgical treatment and to improve outcomes, the screening of patients with a prolonged course for metastases, including those in the thoracic region, is considered to be important.

## 4 Conclusion

If resection without residual cancer is possible in patients with lung metastasis of pancreatic cancer who survive for a long time after curative resection of the primary lesion, a combination of surgical resection and postoperative adjuvant chemotherapy may contribute to improvements in outcomes with the appropriate selection of patients.

## Funding

This research did not receive any specific grant from funding agencies in the public, commercial, or not-for-profit sectors.

## Ethics approval and consent to participate

This study was conducted in accordance with the ethics of the Declaration of Helsinki, and it was approved by the ethics committees of all the facilities involved (No91. in the main facility, Otsu City Hospital).

## Consent for publication

Written informed consent was obtained from the patient for the publication of this case report and any accompanying images.

## CRediT authorship contribution statement

KH, HO, and AM were assigned to surgery and chemotherapy, JT, JI, and AO to chemotherapy and postoperative management, and AM to supervising and directing the research team.

## Declaration of competing interest

None.
